# Proceedings of the Seventeenth Semi-Annual Meeting of the Southwestern Michigan Dental Society

**Published:** 1903-05-15

**Authors:** 


### PROCEEDINGS OF THE SEVENTEENTH SEMI-ANNUAL MEETING OF THE SOUTHWESTERN MICHIGAN DENTAL SOCIETY.

The meeting was called to order by President Warboys Tuesday afternoon, and after prayer by Rev. W. T. Jaques, the mayor of the city made some felicitious remarks and extended to the meeting the cordial greeting of the city of Albion. Dr. S. M. White, of Benton Harbor, accepted the courtesies offered for the society, and suggested that Drs. Warboys, Abell, Dunham and Tower were to be held responsible for this meeting being held in Albion, and therefore logically liable for all damages resulting from it. President Warboys then addressed the meeting and assumed on behalf of the Albion dentists all responsibility for the conduct of the members of the society during the time of the meeting.

### PRESIDENT'S ADDRESS.

It is with great pleasure that I greet you on this the seventeenth semi-annual meeting of this society, as your President, for which honor I must sincerely thank you.

So keen is the competitive spirit of the age, that the advantage of knowledge in the struggle for advancement is apparent to all.

The aim and object of this and all similar meetings is to enable us to be better prepared to combat the destructive efforts of the caries-producing agents that affect the organs of mastication and their surrounding tissues. To acquire more of that precious information, which when absorbed and stored away in a handy place in our memories to be used the moment that it is needed, indeed makes us strong men in our chosen profession. To this end the Secretary and Local Committee have gathered and prepared a feast that we hope will well repay you for the time spent in this assemblage.

This Society was organized July 24th, 1895, in Dr. H. W. Ray's office at St. Joseph, Michigan, with a start of eighteen members, Drs. Ray Backus, Jarvis, Rockwell, Rix, Ellsworth, White, Essig, Codding, Hanson, Burchfield, Funk, Hooper, Runyan, Williamson, Glenn, Cigrand and Rowley, and has held two meetings each year at various cities in the southwestern part of the State.

It was a lusty youngster, for its parents were strong people, so it has grown in eight years from eighteen until now it has over one hundred members in good standing on its roll, all of whom are striving to advance our calling and make us more useful to humanity.

I shall in a brief way call your attention to a few things that will be presented for your consideration and ask you all to enter freely into discussions, for. by this means, we shall receive the benefit of your several thoughts on the various subjects.

The subject of Dental Instruction in the Public Schools is one of great importance and should receive most careful deliberation. Since we have learned that not five percent of the children that attend school are free from caries of the teeth, this subject begins to assume a degree of importance which commands our attention. If, in some way, such a condition can be corrected, we will hail it with delight and shall strive earnestly for its success.

"Variations in Development of Tooth Tissue" is a subject that can not be comprehended too fully in order that we may successfully handle the conditions which are presented for treatment. These conditions have much to do with the welfare of the patients for whom technical operations are performed to correct the progress of caries.

The pathology and treatment of suppurating pulp is a condition which we meet almost daily and which causes untold suffering and distress, which can only be relieved by the dentist, if the tooth is to be retained in the jaw and again made to perform its proper function.

The first permanent molar, its loss or preservation, is just now having considerable attention from regular practitioners as well as specialists in orthodontia. Our patients without it speak of its loss with much regret; those that have it in good condition use it more than any other tooth in the mouth.

When it is necessary to remove the teeth that are worth saving, they may be replanted and made useful for a term of years. This is an exceedingly important subject, and demands close study.

Restoring decayed and broken dental organs to their former contour with a material that will withstand the stress of mastication, be of the same color and not deteriorate under the varied influences that are ever present in the oral cavity and also not permit the recurrence of decay, is what is now justly claimed for poreclain and when we are the masters of this material that we now are for gold, I believe that the latter will find itself resorted to only for very small cavities.

The various clinics can not but be of great interest as they teach by actual observation the way that some one else performs those operations, and the exhibitors of the various instruments and material that are used by us should contriute largely to the success of such meetings as this promises to be. That you may take up these subjects and by carefully considering each one in the light of your own experience, I am sure we shall get some new ideas of practical value and go home with the feeling that our calling is indeed broader than we knew, and demands our frequent assembling together for the purpose of comparing notes, and the establishment of more uniform methods of practice. We shall, I trust, be stimulated to the attainment of a higher order of skill.

